# Supplementary material for: The Evolution and Characterization of the RNA Interference Pathways in Lophotrochozoa
Source: Genome Biol Evol. 2024 May 7;16(5):evae098. doi: 10.1093/gbe/evae098 (PMC11114477; doi:10.1093/gbe/evae098)
Supplement: evae098_Supplementary_Data [file evae098_supplementary_data.zip › Supplementary_materials.zip!/selected_miRNAs/DaRe_selected_miRNas/DaRe_NW_018395069.1_47201.pdf]

Provisional ID	:	NW_018395069.1_47201
Score total	:	228
Score for star read(s)	:	3.9
Score for read counts	:	221.4
Score for mfe	:	1.7
Score for randfold	:	1.6
Score for cons. seed	:	-0.6
Total read count	:	446
Mature read count	:	432
Loop read count	:	0
Star read count	:	14

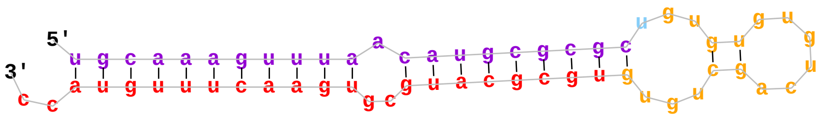

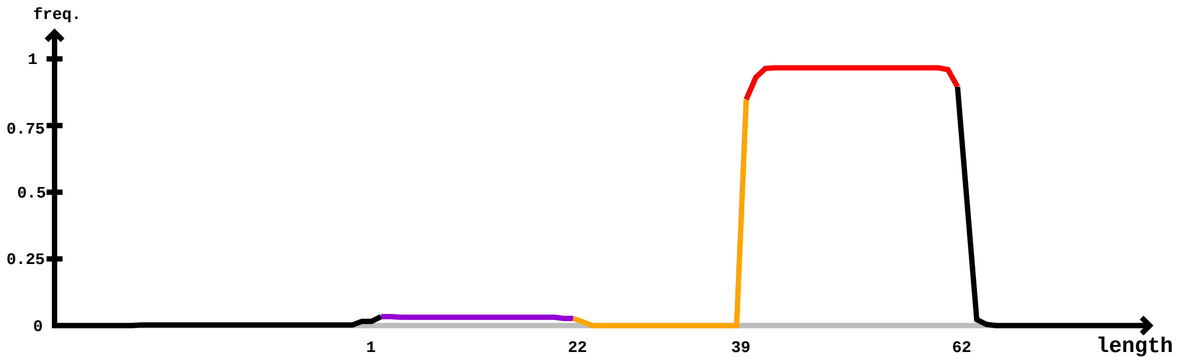


```
                                  Star                                  Mature
5'- uguauccugucacauuugccaugcaaaaacagugcaaaguuuaacaugcgcgcugugugugucagcugugugcgcaugcgugaacuuuguaccgccauugugugugacucau   -3'   obs
    uguauccugucacauuugccaugcaaaaacagugcaaaguuuaacaugcgcgcugugugugucagcugugugcgcaugcgugaacuuuguaccgccauugugugugacucau         exp
    ........(((((((.......((((...(.((((((((((((.(((((((((...((......))...)))))))))..)))))))))))).)...))))))))))).... reads  mm   sample
    ..............................agugcaaaguuuaacaugcgc.............................................................  1      0    Gu1
    ..............................agAgcaaaguuuaacaugcgcgc...........................................................  1      1    Gu1
    ................................ugcaaaguuuaacaugcgcgcu..........................................................  1      0    Gu1
    ......................................................................ugcgcaugcgugaacuuugua.....................  2      0    Gu1
    ......................................................................ugcgcaugcgugaacuuuguac....................  9      0    Gu1
    ......................................................................ugcgcaugcgugaacuuuUuacc...................  1      1    Gu1
    ......................................................................ugcgcaugcgugaacuuuguacU...................  8      1    Gu1
    ......................................................................Cgcgcaugcgugaacuuuguacc...................  1      1    Gu1
    ......................................................................ugcgcaugcgugaacuuuguacc...................  75     0    Gu1
    ......................................................................Ggcgcaugcgugaacuuuguacc...................  1      1    Gu1
    ......................................................................Ngcgcaugcgugaacuuuguacc...................  1      1    Gu1
    ......................................................................ugcgcaugcgugaacuuuguaccU..................  2      1    Gu1
    ......................................................................ugcgcaugcgugaacuuuguaccg..................  1      0    Gu1
    .......................................................................gcgcaugcgugaacuuuguacU...................  11     1    Gu1
    .......................................................................gcgcaugcgugaacuuuguacc...................  1      0    Gu1
    .......................................................................gcgcaugcgugaacuuuguaccC..................  1      1    Gu1
    .......................................................................gcgcaugcgugaacuuuguaccg..................  1      0    Gu1
    ........................................................................cgcaugcgugaacuuuguacc...................  3      0    Gu1
    ........................................................................cgcaugcgugaacuuuguaccg..................  1      0    Gu1
    ........................................................................cgcaugcgugaacuuuguaccgc.................  1      0    Gu1

    ..............................agugcaaaguuuaacaugcgcgc...........................................................  1      0    Ov3
    ................................ugcaaaguuuaacaugcgcgc...........................................................  2      0    Ov3
    ................................ugcaaaguuuaacaugcgcgcu..........................................................  1      0    Ov3
    ......................................................................ugcgcaugcgugaacuuuguacU...................  5      1    Ov3
    ......................................................................ugcgcaugcgugaacuuuguacc...................  53     0    Ov3
    ......................................................................ugcgcaugcguAaacuuuguacc...................  1      1    Ov3
    .......................................................................gcgcaugcgugaacuuuguacU...................  2      1    Ov3
    ........................................................................cgcaugcgugaacuuuguaccgc.................  2      0    Ov3

    ................................ugcaaaguuuaacaugcgcgc...........................................................  2      0    Gu2
    ......................................................................ugcgcaugcgugaacuuuguac....................  8      0    Gu2
    ......................................................................ugcgcaugcgugaacuuuguacA...................  1      1    Gu2
```

```
                                Star                                  Mature
uguauccugucacauuugccaugcaaaaacagugcaaaguuuaacaugcgcgcugugugugucagcugugugcgcaugcgugaacuuuguaccgccauugugugugacucau
......................................................................ugcgcaugcgugaacuuuguacU...................   10   1   Gu2
......................................................................ugcgcaugcgugaacuuuguacc...................   62   0   Gu2
......................................................................ugcgcaugcAugaacuuuguacc...................   1    1   Gu2
......................................................................ugcgcaugcgugaacuuuguaccU..................   3    1   Gu2
.......................................................................gcgcaugcgugaacuuuguac....................   1    0   Gu2
.......................................................................gcgcaugcgugaacuuuguacU...................   5    1   Gu2
.......................................................................gcgcaugcgugaacuuuguacc...................   7    0   Gu2
........................................................................cgcaugcgugaacuuuguaccgU.................   1    1   Gu2
........................................................................cgcaugcgugaacuuuguaccgc.................   2    0   Gu2
.........................................................................gcaugcgugaacuuuguaccgcU................   1    1   Gu2

.......ugucacauuugccaugcaaaaacagug..............................................................................   1    0   Ov2
......................................................................ugcgcaugcgugaacuuuguac....................   3    0   Ov2
......................................................................ugcgcaugcgugaacuuuguacc...................   23   0   Ov2
......................................................................ugcgcaugcgugaacuuuguacU...................   4    1   Ov2
......................................................................ugcgcaugcgugaacuuuguaccU..................   1    1   Ov2
.......................................................................gcgcaugcgugaacuuuguacU...................   1    1   Ov2
.......................................................................gcgcaugcgugaacuuuguacc...................   3    0   Ov2

..............................agugcaaaguuuaacaugcgc.............................................................   1    0   Gu3
..............................agugcaaaguuuaacaugcgcgc...........................................................   1    0   Gu3
..............................agugcaaaguuuaacaugcgcgcu..........................................................   1    0   Gu3
................................ugcaaaguuuaacaugcgcgc...........................................................   1    0   Gu3
......................................................................ugcgcaugcgugaacuuugua.....................   1    0   Gu3
......................................................................ugcgcaugcgugaacuuuguac....................   7    0   Gu3
......................................................................ugcgcaugcgugaacuuuguacc...................   62   0   Gu3
......................................................................ugcgcaugcAugaacuuuguacc...................   1    1   Gu3
......................................................................ugcgcaugcgugaacuuuguacU...................   7    1   Gu3
......................................................................ugcgcGugcgugaacuuuguacc...................   1    1   Gu3
......................................................................ugcgcaugcgugaacuuuguaccU..................   4    1   Gu3
.......................................................................gcgcaugcgugaacuuuguacc...................   1    0   Gu3
.......................................................................gcgcaugcgugaacuuuguacU...................   1    1   Gu3
.......................................................................gcgcaugcgugaacuuuguaccg..................   1    0   Gu3
.......................................................................gcgcaugcgugaacuuuguaccgc.................   1    0   Gu3
........................................................................cgcaugcgugaacuuuguacc...................   1    0   Gu3
........................................................................cgcaugcgAgaacuuuguacc...................   1    1   Gu3
........................................................................cgcCugcgugaacuuuguacc...................   1    1   Gu3
........................................................................cgcaugcgugaacuuuguaccgc.................   1    0   Gu3

................................ugcaaaguuuaacaugcgcgcu..........................................................   1    0   Ov1
......................................................................ugcgcaugcgugaacuuuguac....................   1    0   Ov1
......................................................................ugcgcaugcgugaacuuuguacU...................   1    1   Ov1
......................................................................ugcgcaugcgugaacuuuguacc...................   18   0   Ov1
........................................................................cgcaugcgugaacuuuguaccgcU................   1    1   Ov1
```
